# Olfactory Behavioral Testing in the Adult Mouse

**DOI:** 10.3791/949

**Published:** 2009-01-28

**Authors:** Rochelle M. Witt, Meghan M. Galligan, Jennifer R. Despinoy, Rosalind Segal

**Affiliations:** Department of Pediatric Oncology, Dana Farber Cancer Institute; Department of Neurobiology, Harvard Medical School

## Abstract

The rodent olfactory system is of increasing interest to scientists, studied, in part, in systems biology because of its stereotyped, yet accessible circuitry. In addition, this area's unique ability to generate new neurons throughout an organism's lifetime makes it an attractive system for developmental and regenerative biologists alike. Such interest necessitates a means for a quick, yet reliable assessment of olfactory function. Many tests of olfactory ability are complex, variable or not specifically designed for mice. Also, some tests are sensitive to memory deficits as well as defects in olfactory abilities, confounding obtained results.

Here, we describe a simple battery of tests designed to identify defects in olfactory sensitivity and preference. First, an initial general health assessment allows for the identification of animals suitable for further testing. Second, mice are exposed to various dilutions of scents to ascertain whether there is a threshold difference. Third, mice are presented with various scents, both attractive and aversive, that allow for the assessment of olfactory preference. These simple studies should make the initial characterization of olfactory behavior accessible for labs of varied resources and expertise.

**Figure:**
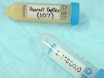


## Protocol

### General Health Exam

A more comprehensive treatment of general health exams can be found in Jacqueline Crawley’s excellent behavioral phenotyping resource, *What’s Wrong with my Mouse?*^1^. We first use a modified observational test battery to assess the general health of the subject. The battery includes items from Samuel Irwin’s comprehensive assessment protocol^2^ to quickly identify those subjects that will be unsuitable for further testing. In brief, the modified battery identifies animals that are physically abnormal (eg. severely under- or over-weight, poorly groomed, dyspneic) or that exhibit abnormal posture, locomotion or behaviors. These all could be manifestations of illness. Any initial evaluation that similarly identifies unhealthy subjects is appropriate.

It is also important to note that animals are used humanely both during the general health exam as well as during subsequent tests. No anesthesia or analgesia is used as these tests do not incorporate any potentially painful procedures.

### Olfactory Sensitivity Testing

This test is designed to detect a difference in olfactory sensitivity between two groups of mice (eg. a wild type and a mutant group of animals). In this test, multiple dilutions of a scent are made. A naïve animal then investigates a single dilution of this scent and the total exploratory time (within 3 minutes) is recorded. Multiple dilutions of the scent are tested on different, naïve subjects and the total exploratory time is used to generate dose response curves for each group of mice. Two researchers should be involved in testing, with one blind to subject genotypes and/or condition.

#### Primary researcher

Obtain four clean cages (8 H x 10 W x 18 L, in inches), all without bedding. Line up the four cages in the biosafety cabinet.
Set up partitions between each arena using opaque filter paper. Place a black poster board across the final partition such that the black side lines the side and bottom of the cage. Cover the top of the arena with a clear piece of plexiglass to allow light to illuminate the arena.(a) 
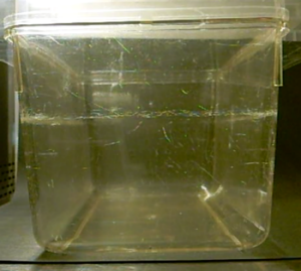
(b)
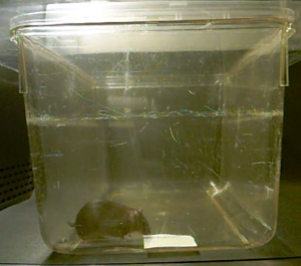
** Figure 1. Arena Set-Up.** (a) Properly draped, empty arena (b) Arena with subject and scented filter paper.
Begin habituating the subjects. Place the first subject in the first cage for habituation. After 15 minutes, move the first subject to the second cage. Clean the first cage with Quatricide TB. Next, place the second subject in the first, cleaned cage. Continue this process with subsequent subjects. Ultimately, each subject should be habituated in all three cages before being placed in the arena (fourth and final cage) for another 15 minutes of habituation prior to testing.

#### Blind researcher

Make an appropriate number of 2 inch by 2 inch squares of filter paper for scent samples.
Using the pre-selected scent, appropriately dilute it. For hydrophobic samples such as peanut butter, use oil as solvent; for all others, use distilled water. Vortex.
Set a digital video recording device at the far end of the biosafety cabinet such that the entire cage end is in focus. Take a preliminary video to ensure parameters are adequate.
Record the order in which dilutions will be tested, taking care to randomize the order of the trials.
Record subject's litter and identification numbers.
Once the subject has been habituated for 15 minutes in the arena, begin preparation for the first trial. Pipette the first dilution up and down in the conical tube before pipetting it onto a filter paper square. The filter paper surface should optimally be ~85% covered (approximately 0.50 mls of solution). Use a kimwipe to make sure the filter paper is not overly saturated.
Begin video recording.
Place the filter paper square in the cage on the opposite end of the animal's current position.
Begin timing.
After 3 minutes, stop the recording and remove the diluted scent.
If only one scent (eg. cinnamon) is to be tested, remove the first test subject from the arena. Repeat steps 6-11 for each animal, appropriately diluting the scent for each subject. If more than one scent is to be tested, however, do not remove the test subject from the arena. Instead, begin timing.
Appropriately dilute the next scent using the same methods as listed above.
After 1 minute, begin video recording. Place the filter paper square with the dilution of the next scent in the cage on the opposite end of the animal's current position.
Repeat steps 6-13 for each scent, noting the video file number for each trial.
Repeat steps 6-14 for each subject. 
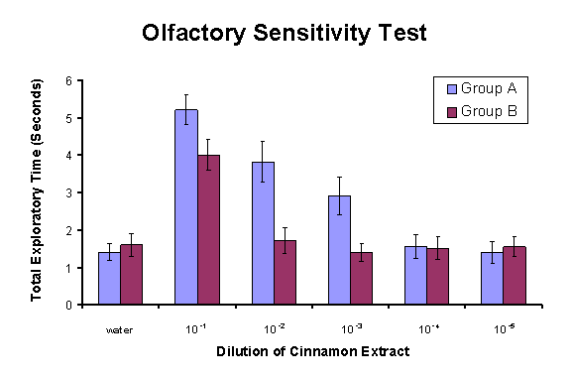
**Figure 2. Representative results for a session of olfactory sensitivity testing.** Each mouse from either the control group (Group A) or the experimental group (Group B) was exposed to one of five different dilutions of cinnamon extract on filter paper for one, three minute session. Total time spent exploring the filter paper was assessed. Mice in Group A have a greater olfactory sensitivity than those in Group B.

### Olfactory Preference Testing

This test is designed to identify specific detection deficiencies, namely the ability to sense attractive or aversive scents. The following procedure borrows liberally from the protocol of Kobayakawa, K., et al.3. Again, two researchers should be involved in testing, with one blind to subject genotypes and/or condition. The total exploratory time is again recorded (upon video viewing) for various scents. Those scents with total exploratory times greater than water are designated as “attractive” while those with times less than water are termed “aversive”. The researcher then considers whether the same scents are “attractive” or “aversive” for different test subjects.

Please follow steps 1-3 in the Olfactory Sensitivity Testing Section for the primary researcher.

#### Blind Researcher

The procedure for the olfactory preference testing is the same, with slight modifications of steps 2 and 4. Please follow steps 1-15 in the Olfactory Sensitivity Testing Section for the blind researcher, with the following changes: For step 2, using the pre-selected scents (a combination of both attractive and aversive scents), make appropriately diluted solutions of each in conical tubes. For hydrophobic samples such as peanut butter, use oil as solvent; for all others, use distilled water. Vortex. For step 4, rather than recording the dilution to be tested, record the order of the varied scents that will be tested, randomizing the order of the trials.

This test is designed to identify specific detection deficiencies, namely the ability to sense attractive or aversive scents. The following procedure borrows liberally from the protocol of Kobayakawa, K. *et al*.^3^. Again, two researchers should be involved in testing, with one blind to subject genotypes and/or condition. The total exploratory time is again recorded (upon video viewing) for various scents. Those scents with total exploratory times greater than water are designated as "attractive", while those with times less than water are termed "aversive". The researcher then considers whether the same scents are "attractive" or "aversive" for different test subjects.

Please follow steps 1-3 in the Olfactory Sensitivity Testing Section for the primary researcher.

#### Blind Researcher

The procedure for the olfactory preference testing is the same, with slight modifications of steps 2, 4 and 13. Please follow steps 1-15 in the Olfactory Sensitivity Testing Section for the blind researcher, with the following changes: For step 2, using the pre-selected scents (a combination of both attractive and aversive scents), make appropriately diluted solutions of each in conical tubes. For hydrophobic samples such as peanut butter, use oil as solvent; for all others, use distilled water. Vortex. For step 4, rather than recording the dilution to be tested, record the order of the varied scents that will be tested, randomizing the order in each trial. For step 13, place the filter paper square with the next scent in the cage on the opposite end of the animal's current position.


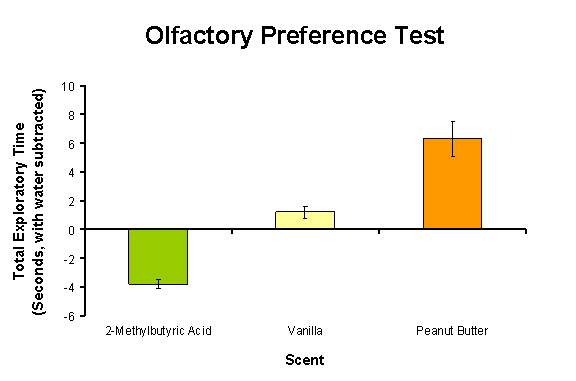


**Figure 3. Representative results for a session of olfactory preference testing.** Each mouse was exposed to each of the different scents on filter paper for one, three minute session. Total time spent exploring the filter paper was assessed. Peanut butter and vanilla are attractive scents (exploratory time_scent_ > exploratory time_water_) and 2-Methylbutyric Acid is aversive (exploratory time_scent_ < exploratory time_water_).

### Scoring of the Olfactory Tests

After acquiring all of the olfactory sensitivity and preference videos, it is necessary to analyze the total time spent exploring each dilution/scent. Have the blind researcher analyze all of the videos for consistency.

Preview the video, noting the following: a) when the filter paper is placed in the arena, b) when the mouse approaches the filter paper, c) when the mouse investigates the filter paper and d) when three minutes have elapsed (from when the filter paper was placed in the arena). Knowing when to focus on the behavior of the mouse makes it easier to accurately score the total time spent exploring upon review.
Review the video. Note the time when the mouse first investigates the filter paper (latency).
Using a stopwatch, time all investigations within three minutes of placing the filter paper in the arena. The time of investigation is defined as any time when the mouse’s nares are < 1 mm from the filter paper. It is important not to include any of the time when the mouse is in close proximity to the filter paper, but is not actively investigating it. Do not include bouts of chewing and licking the paper or bouts of sitting on the paper in the total exploratory time. It is necessary to adhere closely and consistently to these rules to accurately analyze olfactory behavior.
Record latency time, the total time spent investigating the filter paper and video file number.

## Discussion

The aforementioned tests allow for the quick and reliable assessment of basic olfactory function. It is not to be overlooked, however, that this reliability requires the proper selection and preparation of subjects for testing. Animals that are ill or otherwise incapacitated are often inconsistent test subjects. Also, habituation of the test subjects is key. We have found that the use of multiple cages during the habituation process allows the animals to become comfortable with experimenter handling and changing surroundings. We have also found that times shorter than those indicated for habituation are not sufficient to reduce anxiety. Anxious animals also are often unpredictable test subjects and can obscure true differences.

In the actual testing of the mice, it is important not only to be consistent when acquiring the data, but also in analyzing the subjects’ behavior. We have found it useful to only count that time when the test subject’s nose is <1 mm from the filter paper as part of the total exploratory time. Also, behaviors such as biting or chewing the filter paper are not included in the total exploratory time. In addition, latency time (for attractive scents) can also be a useful metric. Different mice backgrounds may require different dilutions of scents for effective discrimination between a control and experimental group. It is also important to sex-match and age-match pairs. We found it helpful to empirically derive appropriate scent dilutions (on subjects similar to the control group) before proceeding with the experimental testing of naïve mice. Finally, these tests were selected to minimize the effect of any other cognitive impairments, most notably, defects in learning and/or memory. To that end, it is critical to use naïve mice for testing.
